# Effect of topiramate on eating behaviours in Prader-Willi syndrome: TOPRADER double-blind randomised placebo-controlled study

**DOI:** 10.1038/s41398-019-0597-0

**Published:** 2019-11-04

**Authors:** Angèle Consoli, Sophie Çabal Berthoumieu, Marie Raffin, Denise Thuilleaux, Christine Poitou, Muriel Coupaye, Graziella Pinto, Said Lebbah, Noel Zahr, Maïthé Tauber, David Cohen, Olivier Bonnot

**Affiliations:** 1 Département de Psychiatrie de l’Enfant et de l’Adolescent, AP-HP, Groupe-Hospitalier Pitié-Salpêtrière, Paris, France; 2 GRC-15, Approche dimensionnelle des épisodes psychotiques de l’enfant et de l’adolescent, Faculté de Médecine, UPMC, Sorbonne Universités, Paris, France; 3 Unité d’Endocrinologie, Obésité, Maladies Osseuses, Génétique et Gynécologie Médicale. Centre de Référence du Syndrome de Prader-Willi, Hôpital des Enfants, Toulouse, France; 4 Assistance-Publique Hôpitaux de Paris (AP-HP), Hopital Marin de Hendaye, French Reference Center for Prader-Willi Syndrome, Hendaye, France; 5 Assistance Publique-Hôpitaux de Paris (APHP), Pitié-Salpêtrière Hospital, French Reference Center for Prader-Willi Syndrome, Nutrition Department, CRNH Ile de France, F-75013 Paris, France; 6 Sorbonne Université, INSERM, Nutriomics team, F-75013 Paris, France; 7 Assistance-Publique Hôpitaux de Paris (AP-HP), Necker Enfants Malades Hospital University Hospital, Pediatric Endocrinology, Diabetology and Gynecology Department, F-75015 Paris, France; 8 Assistance-Publique Hôpitaux de Paris (AP-HP), Pitié-Salpêtrière Hospital, Department of Biostatistics, F-75013 Paris, France; 9 Assistance-Publique Hôpitaux de Paris (AP-HP), Pitié-Salpêtrière Hospital, Department of Pharmacology, F-75013 Paris, France; 10 Axe Pédiatrique du CIC 9302/INSERM. Hôpital des Enfants, Toulouse, France; 11 INSERM U1043, Centre de Physiopathologie de Toulouse Purpan, UPS, Toulouse, France; 12 Institut des Systèmes Intelligents et de Robotiques, CNRS, UMR 7222, UPMC, Sorbonne Universités, Paris, France; 13 Service Universitaire de Psychiatrie de l’Enfant et de l’Adolescent, CHU de Nantes, Nantes, France

**Keywords:** Psychiatric disorders, Clinical genetics

## Abstract

Prader–Willi Syndrome (PWS) is a rare genetic syndrome leading to severe behavioural disorders and mild cognitive impairment. The objective of this double-blind randomised placebo-controlled trial was to study the efficacy and tolerance of topiramate on behavioural disorders in patients with PWS. Participants (aged 12–45 years) had genetically confirmed PWS and severe irritability/impulsivity, eating disorders and/or obesity, and skin picking. Thirty-two participants received a placebo (PBO), and 30 participants received topiramate (TOP) (50–200 mg/day) for 8 weeks. The primary outcome was the rate of responders using the Clinical Global Impression-Improvement (CGI-I) scale. The secondary outcome measures included the Aberrant Behaviour Checklist, the Dykens Hyperphagia Questionnaire (DHK), the Self-Injurious Behaviour Scale (SIBS) and the body mass index (BMI). We found no significant difference in the primary outcome (the CGI-I): 9 (30%) patients were very much or much improved in the TOP group compared to 7 (22.6%) patients in the PBO group. However, the DHK behaviour and severity scores improved significantly more over time in patients treated with topiramate versus those receiving a placebo, with a significant dose–effect relationship. DHK scores were also significantly associated with genetic subtypes and hospitalisation status. The effects of topiramate on eating behaviours remained significant after adjusting for genetic subtype and hospitalisation. Topiramate had therefore a significant effect on eating disorders, with a dose–effect relationship. Given the burden of eating disorders in PWS, we believe that topiramate may become the first psychotropic option within the global care of obesity in individuals with PWS.

## Introduction

Prader-Willi syndrome (PWS) is a rare genetic syndrome occurring in 1/21000 newborns in the general population^1^. This genetic abnormality is located in the 15q11-q13 region, a region with genomic imprinting, and lead to an absence of expression of a paternal gene due to a micro-deletion (65%) or a maternal disomy (30%). Very few cases are due to a defect of the imprinting centre (5%). The clinical presentation is heterogeneous, but some trends can be observed. First, the syndrome follows a developmental course. This is particularly clear for feeding disorders: infants with PWS have poor feeding and social skills along with severe hypotonia that switches to uncontrolled hyperphagia around the age of 2 years. Second, cognitive impairments (mild intellectual disabilities) and psychiatric impairments are common. Third, endocrine dysfunctions are likely to occur via growth hormone (GH) deficiency and hypogonadism. Adults with PWS exhibit numerous challenging behaviours (irritability, aggression, hyperphagia, alimentary compulsions and skin lesions) and psychiatric disorders (mood disorders, obsessive-compulsive disorder, brief psychotic episodes, hallucinations and delusional ideas)^2^. Feeding disorders are severe in 2/3 of cases, and the consequences of them can include obesity, somatic and metabolic disorders and temper outbursts caused by frustration over alimentation or misunderstandings of social situations. Irritability and aggression are particularly challenging for families and institutions. Skin lesions due to skin picking can lead to infections or chronic inflammation and severe anaemia.

The therapeutic approach to PWS has seen tremendous improvements during the last two decades and includes (i) the early use of GHs to improve short stature and body composition^3^, (ii) strict behavioural measures during childhood to control food access and food intake to prevent obesity^4^, and (iii) the possible use of oxytocin for infant feeding deficits, which have shown promising results^5^. However, despite the prevalence of psychiatric disorders and challenging behaviours among those with PWS, to date, no specific treatments have been approved, and very little research has been conducted^6^. Antidepressants (IRS) and antipsychotics seem to be the most frequently used medications^6^. However, antipsychotics can lead to an increase appetite^7^ and are responsible for a worsening of weight gain and irritability as well as temper outbursts related to eating disorders. So far, only two double-blind randomised placebo-controlled studies have been conducted, but they had small sample sizes (*n* = 15). The first trial reported on the efficacy of fenfluramine compared to a placebo on improving eating and aggressive behaviours (skin picking) and inducing weight loss^8^. However, this treatment was withdrawn from the market because of serious cardiac side effects. The second trial reported on the efficacy of rimonabant on eating behaviours compared to a placebo^9^. This treatment was also withdrawn from the market due to its serious psychiatric side effects (anxiety, dysthymia and delusion).

An alternative therapeutic approach has been proposed with topiramate (TOP), an antiepileptic drug that has also been used as an anti-impulsive and mood stabiliser^10^. In addition, this antiepileptic treatment, unlike other ones, does not promote weight gain and instead results in a decrease in appetite and, consequently, weight loss^10^. Topiramate has previously been prescribed for the treatment of eating disorders. In a meta-analysis that pooled five randomised controlled trials of bulimia nervosa (*n* = 128) and binge eating disorder (*n* = 528) patients, topiramate was more efficient in reducing the quantity of binges, the frequency of ‘loss of control’ (binging), and weight compared with a placebo^11^. Two prospective open trials that included 8 patients with PWS each explored the efficacy of topiramate. The first reported an improvement in skin picking and aggressive behaviours but no change in eating behaviours^12^. The second reported improvements in eating behaviours, skin picking and aggressive behaviours, and no side effects were reported^13^. Up to now, no double-blind randomised placebo-controlled trials have been conducted in patients with PWS.

The objective of this double-blind randomised placebo-controlled trial was to study the efficacy of topiramate on eating disorders, skin picking, and irritability/impulsivity in patients with PWS and its tolerance by patients. Given the large spectrum of symptoms, we chose the Clinical Global Impression Scale’s Improvement measure as the primary variable. Secondary variables included symptomatic scales focusing specifically on eating behaviours, skin picking or irritability/impulsivity.

## Methods

### Ethics and regulations

The TOPRADER study was approved by the local ethical committee of the principal investigator (*Comité de Protection des Personnes d’Ile de France VI* under number CPP/104-11). It was also registered with the French regulatory authorities (*Agence Nationale des Produits de Santé* under the number EUDRACT: 2011-003432-32) and the ClinicalTrials.gov international registry under number NCT02810483. All of the subjects received complete information regarding the protocol before enrolment and gave written consent. Regarding minors or adults under guardianship, either the parents or legal guardian also received this information and gave written consent

### Study design

The TOPRADER study was a multicentre double-blind randomised placebo-controlled trial. The objective was to evaluate the efficacy and tolerance of topiramate on irritability/impulsivity, eating disorders and self-mutilation in patients with PWS over an 8-week period. The subjects were randomly allocated into two groups, one taking topiramate, and one taking a placebo. The dosage of topiramate was 50 mg/day initially with increases of 50 mg per week up to 200 mg/day. Visits for inclusion and monitoring occurred at inclusion, baseline and at 2, 4, 6 and 8 weeks (the endpoint).

### Participants

Participants were outpatients and inpatients from the French reference centre for PWS, which encompasses 3 sites (Pitié-Salpêtrière University Hospital in Paris, Marin Hospital in Hendaye and Children University Hospital in Toulouse), and the French reference centre for rare psychiatric disorders (Pitié-Salpêtrière University Hospital in Paris). The inclusion criteria were as follows: a genetically confirmed diagnosis of PWS, being between 12 and 45 years of age inclusive, being over 50 kg in weight and presenting with one of the following symptoms^1^: irritability/impulsivity^2^, eating disorders and/or obesity, or^3^ self-harm. The exclusion criteria were as follows: the presence of hallucinations, meeting the diagnostic criteria for schizophrenia according to the DSM IV, suicidal risk, severe depression, comorbid organic conditions (epilepsy, the use of anticonvulsants or mood stabilisers, unbalanced diabetes (HbA1C > 10%), type 2 diabetes treated with metformin or gibenclamide, a history of nephrolithiasis or glaucoma, hereditary fructose intolerance problems, glucose malabsorption or sucrose-isomaltase insufficiency), current use of an effective dose of topiramate for a sufficient time without efficacy, the introduction or change in dose of a psychotropic medication within the previous 3 months, hypersensitivity to sulphonamides or to any of the components of topiramate or its placebo, the use of a medication with St John’s Wort, pregnancy or breastfeeding, a lack of effective contraception in females of childbearing age, and no informative adult to provide feedback on subjects’ behaviour. Exclusion criteria also included biological abnormalities indicating renal failure (serum creatinine greater than 1.5× normal), hepatic impairment (alanine aminotransferase greater than 2× normal), anaemia (haemoglobin < 12 g/dl (female) or <13 g/dl (male), hyperammonaemia (above laboratory standards), and decreased serum bicarbonates (below laboratory standards).

### Efficacy assessments

The primary outcome measure was the rate of responders to the Clinical Global Impression-Improvement (CGI-I) scale with a response defined as a patient obtaining a score of 1 or 2 (very much or much improved) after 8 weeks of treatment. The CGI-I is a 7-item scale^14^. The secondary outcome measures included^1^ the Aberrant Behaviour Checklist, which includes sub-scores to monitor irritability, lethargy, inappropriate speech, hyperactivity and stereotypies^2,15^; the Dykens Hyperphagia Questionnaire, which includes sub-scores to monitor hyperphagic behaviour, drive and severity^3,16^; the Self-Injurious Behaviour Scale (SIBS), which monitors self-injury behaviours in low-functioning patients with or without autism^17^ (given PWS phenotypes, we focused on self-injurious behaviours to the skin); and^4^ body mass index (BMI), for which weight and size were monitored.

### Safety and tolerability evaluations

Specific attention was given to potential psychiatric adverse effects given that topiramate is known to cause adverse events and that psychiatric symptoms are common in PWS phenotype. At each visit, we monitored patients using the following assessments: the Schizophrenia Positive Symptoms with the Scale for the assessment of positive symptoms^18^, the Brief Psychiatric Rating Scale^19^, the Hamilton Anxiety Scale^20^ and the Columbia Classification Algorithm of Suicide Assessment^21^. Biological parameters were also monitored: NFS, serum electrolytes, creatinine, ammonia plasma, serum bicarbonate, hepatic measures (AST, ALT, and GGT), total fasting ghrelin, fasting glucose, lipid profile, insulin, leptin, triglycerides and HbA1c. We also monitored topiramate plasmatic concentrations at baseline, 4 weeks and 8 weeks to explore a possible dose–effect response in patients exposed to topiramate.

### Statistical analysis

Categorical variables were expressed as frequencies and percentages. Continuous variables were expressed as means (standard deviation, SD) and medians (inter quartiles, IQR).

The comparison of the primary outcome between the 2 treatment groups was performed using a Pearson's chi-squared test. Regarding secondary outcomes, which included continuous variables measured at baseline, 2, 4, 6 and 8 weeks, we performed an analysis of repeated measurements using linear mixed models (LMMs). In this model, the fixed effects were time, treatment groups and group-by-time interaction. We introduced subject as random effect.

We also conducted an exploratory analysis to assess possible modulators such as the study site (Hendaye *vs*. Toulouse vs. Paris), hospitalisation status (inpatient *vs*. outpatient), genetic subtype (disomy *vs*. deletion) and topiramate plasmatic concentration (in µg/mL at baseline, 4 weeks and 8 weeks). We used a logistic regression model for the CGI-I response. Modulators were studied separately using the following formula: [CGI = Treatment + Modulator + Treatment*Modulator]. We used LMMs for secondary continuous variables by adding the effect of the modulator to the model with the following formula: [Secondary variable = Treatment + Time + Treatment *Time + Modulator]. For secondary variables, we only performed the modulator effect analysis in cases of significant effects in group-by-time interactions.

All statistical tests were performed with a 2-tailed α level of 0.05. The data were analysed using R version 3.3.3 (R Core Team, R foundation for Statistical Computing, Vienna, Austria, 2017, URL https://www.R-project.org/).

## Results

### Socio-demographic and clinical characteristics of the sample

**Fig. 1:**
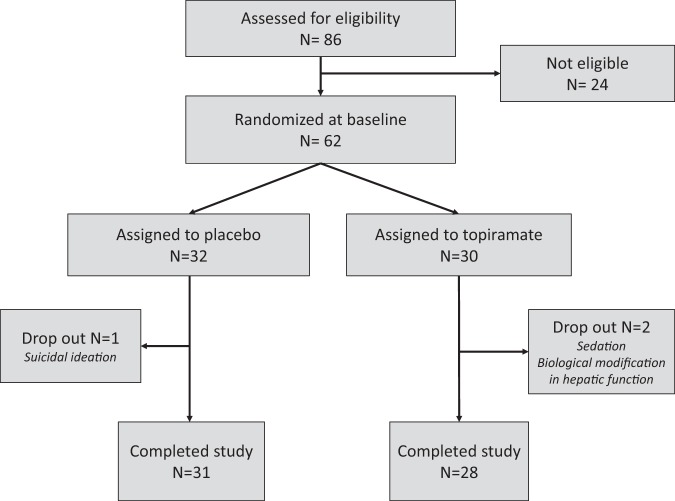
Flowchart of the study

The flowchart of the study is presented in Fig. 1. Between December 2012 and September 2016, 86 subjects were screened for inclusion in this trial. Twenty-four were not eligible or refused to participate. Finally, 62 subjects were randomly allocated to receive topiramate (*n* = 30, TOP) or a placebo (*n* = 32, PBO). Socio-demographic and clinical characteristics of the participants are presented in Table 1. The final randomised sample included 32 females and 30 males, and the mean age was 23.8 (8.3) years [range 12-43]. Of those patients, 33 (53.2%) were outpatients, and 29 (46.8%) were inpatients. Regarding PWS genetic subtypes, 42 (67.7%) subjects had a deletion, 18 (29%) had a maternal disomy and 2 (3.2%) had a defect of the imprinting centre. The mean BMI was 40.74 (12.69), which is categorised as very severely obese (obese class II). At the endpoint, the mean dosage of topiramate in the patients exposed to the active compound was 8.3 (5.83) μg/mL [range: 0.8–22.6].

**Table 1:** Socio-demographic and clinical characteristics of the participants

	Placebo (*n* = 32)	Topiramate (*n* = 30)	Total (*n* = 62)
Gender			
Female *n* (%)	18 (56.2)	14 (46.7)	32 (51.6)
Male *n* (%)	14 (43.8)	16 (53.3)	30 (48.4)
Age			
mean (sd)	23.97 (8.16)	23.7 (8.58)	23.84 (8.3)
median IQR]	25 [15.75–29.25]	24.5 [15.25–31.75]	25 [15.25–1.75]
Genetic subtype			
Deletion *n* (%)	20 (62.5)	22 (73.3)	42 (67.7)
Disomy *n* (%)	11 (34.4)	7 (23.3)	18 (29)
Defect of the imprinting centre *n* (%)	1 (3.1)	1 (3.3)	2 (3.2)
Body mass index (kg/m^2^)			
mean (sd)	41.03 (12.9)	40.43 (12.68)	40.74 (12.69)
median [IQR]	40.2 [32.8–50.65]	36.8 [31.92–49.3]	39.35 [31.92–50.65]
Setting			
Outpatient *n* (%)	16 (50)	17 (56.7)	33 (53.2)
Inpatient *n* (%)	16 (50)	13 (43.3)	29 (46.8)

### Efficacy of topiramate

Regarding the main outcome (CGI-I), we found no significant difference between the number of responders in patients treated with topiramate and in patients treated with the placebo. A total of 9 (30%) patients were very much or much improved in the TOP group compared to 7 (22.6%) in the PBO group (*p* = 0.51).

The secondary outcome variables at baseline and the endpoint are shown in Table 2. For most variables, there was significant improvement over time. However, we found a significant interaction between treatment group and time for the Dykens Hyperphagia Questionnaire behaviour and severity scores, meaning that these scores improved significantly more over time in patients treated with topiramate versus those receiving a placebo (Table 2). The effect of topiramate versus a placebo was also significant using the Hyperphagia Questionnaire for use in PWS Clinical Trials (HQ-CT, data not shown). This recent scale was validated in PWS during our trial and only differs from the Dykens Hyperphagia Questionnaire by the removal of 3 items^22^. There was no significant interaction between group and time for any other secondary variable (sub-scores of the ABC and SIBS-skin picking) except for the ABC-lethargy sub-score: although lethargy improved in both groups, it appeared that the decrease was significantly lower over time in the TOP group versus the PBO group.

**Table 2:** Changes in clinical characteristics from baseline to the final visit (8 weeks)

	Placebo (*n* = 32)		Topiramate (*n* = 30)		Test	*p*
	Baseline	8 weeks	Baseline	8 weeks		
	Primary variable					
CGI-I: *n* (%) much and very much improved		7 (22.6%)		9 (30%)	Pearson Chi-squared	0.51
	Secondary variables:mean (SD)					
Dykens, Behaviour	12.88 (4.08)	11.6 (4.11)	13.37 (4.23)	10 (3.88)	ß topi: 1.47 ß time: –.297 **ß inter:** –**.39**	0.18 0.005 **0.011**
Dykens, Drive	11.16 (3.61)	9.83 (3.53)	11.87 (4.13)	8.96 (4.39)	ß topi: 1.61 ß time: –.343 ß inter: –.292	0.163 0.006 0.1
Dykens, Severity	4.81 (2.13)	4.3 (1.64)	5.17 (2.46)	3.82 (2.16)	ß topi: 0.88 ß time: –.076 **ß inter: –.233**	0.14 0.21 **0.007**
BMI, kg/m2	41.03 (12.9)	40.46 (11.38)	40.43 (12.68)	38.66 (11.14)	ß topi: –.259 ß time: –8.125 ß inter: 1.4	0.8 < 0.001 0.16
BPRS	27.59 (10.46)	27.2 (9.55)	27.53 (10.96)	26.54 (12.73)	ß topi: –.929 ß time: –.337 ß inter: .025	0.75 0.026 0.91
ABC, Irritability	10.28 (7.88)	4.73 (5.51)	9.7 (7.28)	4.71 (6.62)	ß topi: –1.11 ß time: –1.23 ß inter: .277	0.55 < 0.001 0.39
ABC, Lethargy	6.47 (7.32)	3.47 (4.88)	5.5 (6.06)	4.25 (4.91)	ß topi: –1.48 ß time: –.727 ß inter: **.506**	0.34 < 0.001 **0.021**
ABC, Stereotype	0.66 (1.64)	0.67 (1.81)	1.27 (3.62)	1.18 (3.61)	ß topi: .525 ß time: –.023 ß inter: –.009	0.46 0.62 0.89
ABC, Hyperactivity	4.44 (5.32)	2.3 (3.11)	6.2 (8.79)	4.64 (7.65)	ß topi: 1.59 ß time: –.426 ß inter: .043	0.33 0.001 0.82
ABC, Inappropriate speech	2.28 (3.21)	1.5 (2.32)	2.13 (2.92)	1.5 (2.77)	ß topi: –.016 ß time: –.158 ß inter: .031	0.98 0.015 0.74
SIBS, Skin picking	7.69 (5.89)	5.67 (3.87)	9.17 (5.84)	7 (5.46)	ß topi: .584 ß time: –.528 ß inter: .11	0.69 < 0.001 0.61

*CGI* Clinical Global Impression, *ABC* Aberrant Behaviour Checklist, *SIBS* Self-Injurious Behaviour Scale, *BMI* Body mass index, *BPRS* Brief Psychiatric Rating Scale, *ß topi*: ß topiramate, *ß inter*: ß topiramate * time.

The ß coefficients were the regression coefficients of the LMM

Finally, a trend was observed for a decrease in BMI in the topiramate group versus the placebo group (40.4 to 38.7 in the TOP group *vs*. 41.0 to 40.5 in the PBO group), but without a significant effect for the interaction between time and group in the statistical model (see Table 2).

### Exploratory analysis regarding modulators

As explained in the Methods section, we aimed to explore 4 possible modulators of the topiramate response: the study site (Hendaye *vs*. Toulouse vs. Paris), hospitalisation status (inpatient *vs*. outpatient), genetic subtype (disomy *vs*. deletion) and topiramate plasmatic concentration (in µg/mL at baseline, 4 weeks and 8 weeks). Detailed analyses are reported in the supplemental materials (table S1 to S12). For the CGI response, we found no modulating effect of the variables we explored. For the Dykens Hyperphagia Questionnaire behaviour and severity scores and the ABC-lethargy sub-score, we found four significant modulator effects. The Dykens Hyperphagia Questionnaire behaviour score was significantly associated with the genetic subtype, with patients with disomy showing lower scores than patients with a deletion (ß estimate = −2.15, *p* = 0.011). The Dykens Hyperphagia Questionnaire severity score was significantly associated with the hospitalisation status and study site. Inpatients had lower scores than outpatients (ß estimate = −1.11, *p* = 0.017), and patients in Hendaye had lower scores than patients at other sites (ß estimate = −1.62, *p* = 0.002). To explore the modulating effect of the topiramate plasmatic concentration, we only used the HQ-CT score, since the models only included participants exposed to topiramate. Patients with higher topiramate plasmatic concentrations had lower scores for eating disorder behaviours (ß estimate = −0.32, *p* = 0.0029). The effect remained significant after adjusting for BMI, genetic subtype and study site (ß estimate = −0.31, *p* = 0.0032; ß estimate = −0.32, *p* = 0.003; ß estimate = −0.33, *p* = 0.002, respectively). Finally, the ABC-lethargy sub-score was significantly associated with the hospitalisation status; inpatients had lower scores than outpatients (ß estimate = −3.06, *p* = 0.004).

### Safety

Adverse events are shown in Table 3. There were only three cases of severe adverse events leading to a patient’s withdrawal: 1 for suicidal ideation in the PBO group, 1 for hepatic dysfunction and 1 for excessive sedation in the TOP group. Overall, 18 subjects presented with at least one adverse event, and the total number of adverse events was 23. We observed 7 biological modifications in hepatic function, 6 cases of hyperammonaemia, 1 skin rash, 2 infectious episodes, 4 cases of sedative effects or psychomotor slowdowns, 1 hospitalisation for a suicidal attempt, 1 case of anxiety and tears and 1 case of suicidal ideation. Of these, the following 14 were reported as occurring in the TOP group: 4 cases of sedative effects or psychomotor slowdowns, 4 biological modifications in hepatic function, 4 cases of hyperammonaemia, and 2 infectious episodes (bronchitis asthma and sinusitis). This low number of AEs did not allow for a statistical analysis. However, it appeared that most of the AEs occurred in both groups, except for sedation, which occurred only in the TOP group. We found no changes in Schizophrenia Positive Symptoms and the Hamilton Anxiety Scale.

**Table 3:** Adverse events (*n* = 23)

	Placebo (*n* = 9)	Topiramate (*n* = 14)	Total (*n* = 23)
Sedative effects or psychomotor slowdowns	0	4 (28.6%)	4 (17.4%)
Anxiety and tears	1 (11%)	0	1 (4.3%)
Suicidal ideation	1 (11%)	0	1 (4.3%)
Hospitalisation (suicidal attempt)	1 (11%)	0	1 (4.3%)
Biological modifications in hepatic function	3 (33.3%)	4 (28.6%)	7 (30.4%)
Hyperammonaemia	2 (22%)	4 (28.6%)	6 (26%)
Skin rash	1 (11%)	0	1 (4.3%)
Infectious episode	0	2 (14.2%)	2 (8.7%)

## Discussion

The TOPRADER study is the first randomised placebo-controlled trial assessing improvement of behavioural impairments in PWS. There was no significant effect in the topiramate group compared to the placebo group on our primary outcome, the CGI-I. However, the results strongly support that topiramate is significantly effective on hyperphagia and challenging eating behaviour but not on the other behavioural dimensions. The Clinical Global Impression scale is a global scale that covers eating behaviours, irritability/aggression and skin picking in PWS patients. The finding that the effect of topiramate was significant for eating behaviours but not for the other dimensions may explain why the CGI was not significant between the two groups. Indeed, an important result from the study is the significant effect of topiramate on reducing challenging eating disorders in PWS patients combined with overall good short-term safety.

Up to now, only one prospective open trial reported improvements in eating behaviours^13^ in PWS, but it was not a double-blind randomised placebo-controlled trial. Eating disorders are very challenging in PWS. It has been found that controlling food intake is very effective when established early on^1,4^. However, very few data exist concerning pharmacological therapeutic approaches^6^. In this trial, the significant positive effects of topiramate over time on eating behaviours in PWS was observed in two sub-scores of the Dykens Hyperphagia Questionnaire^16^: hyperphagic behaviours and their severity. These hyperphagic behaviours are the most challenging behaviours in PWS. Indeed, they start early (6 years old in our sample), and their consequences are dramatic both physically and psychologically through obesity (and its morbidity) and behavioural disorders due to alimentary frustrations (irritability, aggression and temper outbursts)^2,23^. These behaviours can frequently lead to exclusion from institutions and an escalation in the use of pharmacological drugs with potential secondary aggravating effects (e.g., second-generation antipsychotics are frequently used and can lead to increased appetite and metabolic disorders).

Regarding improvements in eating disorder severity over time, we found two interesting modulator effects. To interpret our results, it is important to note that study sites and hospitalisation status were highly correlated since the Hendaye site is a hospitalisation unit dedicated to PWS patients and proposing systematic behavioural food control. The reduction in eating behaviour severity was greater when there was a concomitant effect from topiramate treatment and behavioural/educational methods through hospitalisation. The second interesting significant modulation was the dose-response effect we observed in patients exposed to topiramate. Patients with higher topiramate plasmatic concentrations had lower Dykens Hyperphagia Questionnaire behaviour scores. The effect remained significant after adjusting for BMI, genetic subtype and study site. Although we did not find a significant effect on other challenging behaviours and BMI, it is likely that the positive effect on eating behaviours should also have a positive effect on temper outbursts related to food frustrations and BMI in the longer term. The fact that the trial lasted 8 weeks, with only 5 weeks with full posology, can explain the absence of a significant effect over time for BMI. Longer follow-up studies are warranted.

Regarding safety, 23 AEs were reported, including 14 AEs among patients receiving topiramate. Concerning psychiatric events, sedative effects or psychomotor slowdowns were reported in four patients (28.6%). These data are confirmed by lethargy over time as measured by the ABC scale, which was significantly increased in patients in the TOP versus the placebo group. Other adverse events in patients with topiramate concerned biological (hyperammonaemia, modifications in hepatic function) and organic disorders (infectious episodes). No isolated fever was reported. In PWS, thermal dysregulation with fever can be observed and hypohidrosis and hyperthermia were described in a few case reports of patients treated by topiramate^24^. We should thus be careful about this possible side effect in this population.

In the topiramate group, only two patients dropped out of the study (for excessive sedation and hepatic dysfunction). This pharmacological treatment seems to be well tolerated in the short term, especially with regard to psychiatric side effects; no hallucinations were reported. Suicidal ideation (*n* = 1) and an attempt (*n* = 1) were reported in the placebo group. Regarding biological side effects, regular biological monitoring should be performed, as with most antiepileptic drugs, with particular attention paid to ammonia levels, as they can induce long-term neurotoxicity. However, ammonia levels can fluctuate and be difficult to measure, particularly with temperature sensitive dosages. This may explain the cases of hyperammonaemia observed in the placebo group. Up to now, only case reports of encephalopathy induced by the interaction of valproate and topiramate have been reported in the literature^25^.

The results of this study should be interpreted considering both its strengths and limitations. The trial strengths include it being the first double-blind randomised placebo-controlled study of PWS, the reporting of a substantial sample (*n* = 62) considering that PWS is a rare disease, and the use of the Dykens Hyperphagia Questionnaire behaviour scale, which is commonly used and pertinent in the PWS population^26,27^. The trial also has several limitations: first, we chose a primary outcome measure that was overly broad, the CGI-I. This was based on the limited literature regarding PWS psychopharmacology. We balanced this with the literature on topiramate that retains two main indications: epilepsy^28^ and bulimia nervosa/binge eating^11^. Second, the study was short in duration (only 8 weeks, with only 5 at a stable posology). Therefore, we may have missed some long-term efficacy effects as well as long-term AEs. Specifically, we cannot exclude that the reassuring absence of hallucinations and severe psychiatric symptomatology is due to ending the study before the occurrence of such AEs. A longer safety study should be conducted to monitor topiramate use in PWS.

## Conclusions

PWS is a rare developmental syndrome that includes cognitive impairment, eating and behavioural disorders, learning disabilities and an increased prevalence of psychiatric disorders. This first double-blind randomised placebo-controlled trial reported a significant efficacy of topiramate on eating disorders. Given the burden of eating disorders in PWS, we believe that this effect on eating disorders may offer new therapeutic opportunities for individuals with PWS.

## Supplementary information

Supplementarymaterials
