# Supplementary material for: Effect of topiramate on eating behaviours in Prader-Willi syndrome: TOPRADER double-blind randomised placebo-controlled study
Source: Transl Psychiatry. 2019 Nov 4;9:274. doi: 10.1038/s41398-019-0597-0 (PMC6828670; doi:10.1038/s41398-019-0597-0)
Supplement: Supplementary file 1 — Supplementarymaterials [file 41398_2019_597_MOESM1_ESM.docx]

Table S1. Interaction between hospitalization and treatment on CGI

|  | **Coefficient** | **OR** | **CI95** | **p_values** |
| --- | --- | --- | --- | --- |
| Treatment | 0.51 | 1.67 | 0.32 - 8.59 | 0.54 |
| Hospitalization | 0.29 | 1.33 | 0.24 - 7.28 | 0.74 |
| Interaction : Treatment* Hospitalization | -0.22 | 0.8 | 0.08 - 8.1 | 0.85 |

[Model: CGI = Treatment + Hospitalization + Treatment* Hospitalization]

Table S2. Interaction between study site and treatment on CGI

|  | **Coefficient** | **OR** | **CI95** | **p_values** |
| --- | --- | --- | --- | --- |
| topiramate | 0.85 | 2.33 | 0.31 - 17.55 | 0.41 |
| study siteHendaye | 0.15 | 1.17 | 0.17 - 8.09 | 0.88 |
| study siteToulouse | -0.36 | 0.7 | 0.05 - 10.01 | 0.79 |
| topiramate*study siteHendaye | -0.56 | 0.57 | 0.04 - 7.66 | 0.67 |
| topiramate*study siteToulouse | -1.03 | 0.36 | 0.01 - 13.44 | 0.58 |

## [Model: CGI = Treatment + Study site + Treatment*Study site]

Table S3. Interaction between genetic status and treatment on CGI

|  | **Coefficient** | **OR** | **CI95** | **p_values** |
| --- | --- | --- | --- | --- |
| Treatment | 0.12 | 1.12 | 0.28 - 4.47 | 0.8672 |
| Genetic subtype: Disomy | -0.29 | 0.75 | 0.12 - 4.77 | 0.7606 |
| Interaction: Treatment * Genetic subtype | 0.98 | 2.67 | 0.21 - 34.45 | 0.4524 |

[Model: CGI = Treatment + Genetic status+ Treatment*Genetic status]

Table S4. Interaction between hospitalization and treatment on Dykens Behaviour

|  | **Estimate** | **Std..Error** | **t.value** | **p.z** |
| --- | --- | --- | --- | --- |
| Intercept | 13.33 | 0.90 | 14.87 | 0.000 |
| Treatment | 1.45 | 1.10 | 1.31 | 0.189 |
| Time | -0.30 | 0.11 | -2.79 | 0.005 |
| hospitalization | -0.24 | 0.93 | -0.26 | 0.186 |
| Interaction: Treatment * Time | -0.39 | 0.15 | -2.55 | 0.011 |

[Model: Dykens Behaviour = Treatment + Time + Treatment*Time + hospitalization]

Table S5. Interaction between study site and treatment on Dykens Behaviour

|  | **Estimate** | **Std..Error** | **t.value** | **p.z** |
| --- | --- | --- | --- | --- |
| Intercept | 14.22 | 1.04 | 13.69 | 0.000 |
| Treatment | 1.43 | 1.09 | 1.31 | 0.192 |
| Time | -0.30 | 0.11 | -2.80 | 0.005 |
| Study site Hendaye | -1.12 | 1.06 | -1.06 | 0.291 |
| Study site Toulouse | -2.08 | 1.26 | -1.65 | 0.100 |
| Interaction: Treatment * Time | -0.39 | 0.15 | -2.55 | 0.011 |

[Model: Dykens Behaviour = Treatment + Time + Treatment*Time + Study site]

Table S6. Interaction between genetic status and treatment on Dykens Behaviour

|  | **Estimate** | **Std..Error** | **t.value** | **p.z** |
| --- | --- | --- | --- | --- |
| Intercept | 13.83 | 0.83 | 16.73 | 0.000 |
| Treatment | 1.69 | 1.08 | 1.55 | 0.120 |
| Time | -0.29 | 0.11 | -2.71 | 0.007 |
| **Genetic subtype: Disomy** | **-2.15** | **0.98** | **-2.19** | **0.011** |
| Interaction: Treatment * Time | -0.43 | 0.15 | -2.78 | 0.005 |

[Model: Dykens Behaviour = Treatment + Time + Treatment*Time + Genetic status: Disomy]

Table S7. Interaction between hospitalization and treatment on Dickens Severity

|  | **Estimate** | **Std..Error** | **t.value** | **p.z** |
| --- | --- | --- | --- | --- |
| Intercept | 5.44 | 0.47 | 11.62 | 0.000 |
| Treatment | 0.80 | 0.58 | 1.38 | 0.167 |
| Time | -0.08 | 0.06 | -1.24 | 0.213 |
| **hospitalization** | **-1.11** | **0.47** | **-2.35** | **0.017** |
| Interaction: Treatment * Time | -0.23 | 0.09 | -2.72 | 0.007 |

[Model: Dykens Severity = Treatment + Time + Treatment*Time + hospitalization]

Table S8. Interaction between study site and treatment on Dickens Severity

|  | **Estimate** | **Std..Error** | **t.value** | **p.z** |
| --- | --- | --- | --- | --- |
| Intercept | 5.96 | 0.54 | 11.12 | 0.000 |
| Treatment | 0.79 | 0.57 | 1.38 | 0.168 |
| Time | -0.08 | 0.06 | -1.26 | 0.208 |
| **Study site Hendaye** | **-1.62** | **0.54** | **-3.03** | **0.002** |
| Study site Toulouse | -1.21 | 0.64 | -1.89 | 0.059 |
| Interaction: Treatment * Time | -0.23 | 0.09 | -2.71 | 0.007 |

[Model: Dykens Severity = Treatment + Time + Treatment*Time + Study site]

Table S9. Interaction between genetic status and treatment on Dickens Severity

|  | **Estimate** | **Std..Error** | **t.value** | **p.z** |
| --- | --- | --- | --- | --- |
| Intercept | 5.03 | 0.46 | 10.91 | 0.000 |
| Treatment | 0.98 | 0.60 | 1.61 | 0.107 |
| Time | -0.07 | 0.06 | -1.17 | 0.240 |
| Genetic subtype: Disomy | -0.70 | 0.54 | -1.28 | 0.133 |
| Interaction: Treatment * Time | -0.25 | 0.09 | -2.82 | 0.005 |

[Model: Dykens Severity = Treatment + Time + Treatment*Time + Genetic status: Disomy]

Table S10. Interaction between hospitalization and treatment on ABC: Lethargy

|  | **Estimate** | **Std..Error** | **t.value** | **p.z** |
| --- | --- | --- | --- | --- |
| Intercept | 8.73 | 1.21 | 7.22 | 0.000 |
| Treatment | -1.68 | 1.50 | -1.12 | 0.264 |
| Time | -0.72 | 0.15 | -4.72 | 0.000 |
| **hospitalization** | **-3.06** | **1.23** | **-2.49** | **0.004** |
| Interaction: Treatment * Time | 0.50 | 0.22 | 2.29 | 0.022 |

[Model: ABC: Lethargy = Treatment + Time + Treatment*Time + hospitalization]

Table S11. Interaction between study site and treatment on ABC: Lethargy

|  | **Estimate** | **Std..Error** | **t.value** | **p.z** |
| --- | --- | --- | --- | --- |
| Intercept | 7.82 | 1.41 | 5.56 | 0.000 |
| Treatment | -1.65 | 1.50 | -1.10 | 0.270 |
| Time | -0.72 | 0.15 | -4.72 | 0.000 |
| Study site Hendaye | -2.17 | 1.42 | -1.53 | 0.127 |
| Study site Toulouse | 2.12 | 1.69 | 1.25 | 0.212 |
| Interaction: Treatment * Time | 0.50 | 0.22 | 2.28 | 0.022 |

[Model: ABC: Lethargy = Treatment + Time + Treatment*Time + study site]

Table S12. Interaction between genetic status and treatment on ABC: Lethargy

|  | **Estimate** | **Std..Error** | **t.value** | **p.z** |
| --- | --- | --- | --- | --- |
| Intercept | 7.03 | 1.22 | 5.78 | 0.000 |
| Treatment | -1.28 | 1.60 | -0.80 | 0.421 |
| Time | -0.73 | 0.16 | -4.69 | 0.000 |
| Genetic subtype: Disomy | 0.70 | 1.45 | 0.48 | 0.094 |
| Interaction: Treatment * Time | 0.50 | 0.22 | 2.23 | 0.026 |

[Model: ABC: Lethargy = Treatment + Time + Treatment*Time + Genetic status: Disomy]
